# Performance of rapid diagnostic tests, microscopy, and qPCR for detection of Plasmodium parasites among community members with or without symptoms of malaria in villages located in North-western Tanzania

**DOI:** 10.1186/s12936-025-05361-2

**Published:** 2025-04-09

**Authors:** Rule Budodo, Salehe S. Mandai, Catherine Bakari, Misago D. Seth, Filbert Francis, Gervas A. Chacha, Angelina J. Kisambale, Daniel P. Challe, Daniel A. Petro, Dativa Pereus, Rashid A. Madebe, Ruth B. Mbwambo, Ramadhan Moshi, Sijenunu Aaron, Daniel Mbwambo, Abdallah Lusasi, Stella Kajange, Samwel Lazaro, Ntuli Kapologwe, Celine I. Mandara, Deus S. Ishengoma

**Affiliations:** 1 National Institute for Medical Research, Dar es Salaam, Tanzania; 2 National Institute for Medical Research, Tanga Research Centre, Tanga, Tanzania; 3 University of Dar es Salaam, Dar es Salaam, Tanzania; 4 Muhimbili University of Health and Allied Sciences, Dar es Salaam, Tanzania; 5 National Malaria Control Programme, Dodoma, Tanzania; 6 President’s Office, Regional Administration and Local Government, Dodoma, Tanzania; 7 Directorate of Preventive Services, Ministry of Health, Dodoma, Tanzania; 8 Department of Biochemistry, Kampala International University in Tanzania, Dar es Salaam, Tanzania

**Keywords:** Rapid diagnostic tests, Microscopy, qPCR, malaria, Artemisinin partial resistance, Northwestern Tanzania

## Abstract

**Background:**

Despite the implementation of different control interventions, *Plasmodium* parasite infections in the communities (among asymptomatic and symptomatic individuals) still play a crucial role in sustaining malaria transmission. This study evaluated the performance of rapid diagnostic tests (RDTs), microscopy, and quantitative PCR (qPCR) in detecting *Plasmodium* parasites among community members in five villages of Kyerwa district, Kagera region in north-western Tanzania.

**Methods:**

The study used samples and data collected during a community cross-sectional survey of asymptomatic and symptomatic participants (n = 4454) aged ≥ 6 months which was conducted in July and August 2023. *Plasmodium* parasites were detected using RDTs, microscopy, and qPCR (targeting 18S rRNA gene). The performance of RDTs and microscopy was assessed by sensitivity, specificity, and predictive values, using qPCR as the reference method. Factors affecting the accuracy of these methods were determined using a multivariate logistic regression model.

**Results:**

The prevalence of *Plasmodium* parasite infections among 4454 participants was 44.4%, 32.1%, and 39.8% by RDTs, microscopy, and qPCR, respectively. The prevalence of *Plasmodium falciparum*, *Plasmodium malariae* and *Plasmodium ovale* mono-infection by microscopy was 28.7%, 0.2%, and 0.3%, while by qPCR it was 35.3%, 0.4% and 0.5%, respectively. The geometric mean parasite densities (GMPDs) by microscopy were 642 (95% confidence intervals (CI) = 570–723), 126 (95% CI = 98–162), and 124 (95% CI = 82–160) asexual parasites/µL for *P. falciparum, P. ovale spp.*, and *P. malariae*, respectively. By qPCR, the GMPDs were 1180 (95% CI = 1032–1349) parasites/µL for *P. falciparum,* 44 (95% CI = 32–61) for *P. ovale* spp., and 50 (95% CI = 29–89) for *P. malariae*. The sensitivity and specificity of RDTs were 94.0% (95% CI = 92.8–95.1%) and 87.5% (95% CI = 86.2–88.7%), respectively, whereas those of microscopy were 74.6% (95% CI = 72.5–76.6%) and 95.2% (95% CI = 94.3–96.0%), respectively. The sensitivity of RDTs, and microscopy was low at very low parasitaemia (< 100 parasites/μL) but increased significantly with increasing parasitaemia, reaching ≥ 99.6% at > 10,000 parasites/μL (p < 0.001).

**Conclusion:**

High prevalence of *Plasmodium* parasites was detected, and the performance of RDTs and qPCR was comparable, but microscopy had lower performance. Higher sensitivity of RDTs compared to microscopy indicates that RDTs are effective for detection of infections caused by *Plasmodium* parasites in routine case management and surveillance in this area with confirmed artemisinin partial resistance (ART-R) and can be utilized in the ongoing plans to develop a response to ART-R.

## Background

Malaria remains a major public health threat, especially among under-fives and pregnant women in sub-Saharan Africa (SSA), including Tanzania [1]. In 2022, 93.6% of global malaria cases and 95.4% of the deaths due to malaria were reported in the World Health Organization’s (WHO) African region (WHO—Afro), where 78.1% of all malaria deaths in this region were among under-fives [2]. Tanzania is among the countries in SSA and WHO-Afro region with the highest burden of malaria; it reported 4.4% of all global malaria deaths in 2022 [2]. Malaria in Tanzania and most of other SSA countries is caused by three species of *Plasmodium* parasites: *Plasmodium falciparum, Plasmodium ovale* and *Plasmodium malariae,* with the majority of the infections (> 85%) caused by *P. falciparum* [3–6]. Malaria infections due to *Plasmodium vivax* have been sporadically reported, but this species is less prevalent due to the absence of Duffy antigen (among African populations), the binding site for *P. vivax* [7]. Despite the efforts that have been made to control and eliminate malaria in Tanzania, challenges such as the emergence and spread of insecticide resistance in the vectors [8], antimalarial-resistant *P. falciparum* [9], histidine-rich protein 2/3 (*hrp2/3*) gene deletions [10], and the emergence of invasive *Anopheles stephensi* vectors threaten the progress made in the past two decades [11].

For effective case management, WHO recommends parasitological confirmation of all suspected malaria cases by rapid diagnostic tests (RDTs) or microscopy before initiating treatment with artemisinin-based combination therapy (ACT) [12]. Currently, RDTs are the primary diagnostic tool in Tanzania and are used for diagnosis of malaria by detecting *Plasmodium* parasites’ antigens [13]. The wide use of RDTs is due to simplicity, short turnaround time, limited infrastructure requirements, and cost-effectiveness [14]. However, the performance of RDTs is affected by various factors, such as storage conditions, parasitemia, type of antigen, and operator skills [15]. Studies show that most RDTs often have reduced sensitivity at low parasite densities, such as parasitaemia less than 100 asexual parasites/μL. As a result, RDTs may also fail to detect low-density and chronic latent infections, especially in asymptomatic populations, particularly in low-transmission settings [16, 17]. The currently used RDTs are based on the detection of three antigens, which include lactate dehydrogenase (LDH), Aldolase, and *P. falciparum* histidine-rich protein 2 (*pf*HRP2) [18]. Although *pf*HRP2-based RDTs are widely used due to their sensitivity, stability, and abundance, their accuracy may be limited by different factors such as mutations or *hrp2/3* gene deletions [10] and/ or prozone effect [19, 20], thus leading to false negative results [15].

For more than a century, microscopy has remained the gold standard for diagnosis of human malaria parasites due to its ability for detection and visualization, differentiation of parasite species, detection of parasite stages (sexual or asexual forms) and quantification of malaria parasites in blood smears [21]. While microscopy offers high specificity and the ability to determine parasite species and quantify parasitemia, the sensitivity of microscopy can be influenced by factors such as the quality of staining reagents and the skills of microscopists [22]. The limit of detection (LOD) of the expert microscopist can be as low as five (5) parasites/μL, while the average LOD for most microscopists ranges from 50 to 100 parasites/μL [23]. Other limitations of microscopy include the demand for high-quality microscopes which are well maintained, a laboratory facility, reliable electricity, and reagents (fixing and staining reagents, and filtered water at the correct pH) [22, 24]. Due to these limitations, most malaria-endemic countries deployed RDTs and have been using them for parasitological diagnosis of malaria by confirmation of infections by *Plasmodium* parasites. This has been critical for supporting prompt and effective treatment using ACT, which significantly contributed to reducing the burden of malaria over the past two decades.

Malaria diagnosis by molecular techniques or nucleic acid detection methods is a highly sensitive and specific method that detects, amplifies, and quantifies DNA specific to the target  parasites [25]. Different methods, such as quantitative PCR (qPCR) have been developed and utilized for the detection of  *Plasmodium* parasites. Nucleic acid detection methods offer accurate and rapid detection of different parasite species, even at low parasite densities (1–5 asexual parasites/μL), and can accurately differentiate different *Plasmodium* species, as well as identify mixed or complex infections [16, 26, 27]. These methods have high sensitivity and specificity which enable them to identify infections in different groups including asymptomatic reservoirs that are missed by conventional diagnostic methods (microscopy and RDTs), and can provide a more accurate assessment of malaria prevalence within a population [4, 28]. Additionally, molecular techniques facilitate the detection and monitoring of anti-malarial drug resistance by detecting genetic markers associated with resistance. They are also useful in efficacy studies of anti-malarial drugs for differentiating recrudescent from new infections and thus establishing the efficacy of anti-malarials [29]. Because they have some major limitations such as infrastructure requirements, lack of skilled experts, and high purchasing and operational costs, nucleic acid detection methods have not been widely used in malaria-endemic countries including Tanzania [30].

In areas with high transmission, *Plasmodium* parasite infections in the communities (mainly among asymptomatic individuals) play an important role in malaria transmission [31]. These individuals who remain in the community without seeking for and receiving health care harbour parasite infections, more often with low levels of parasitaemia that are difficult to detect by conventional diagnostic methods like RDTs and microscopy. This makes the detection and targeting of these community infections difficult [15, 32]. In addition, there is a paucity of data on how routine diagnostic methods such as RTDs and microscopy perform in the detection of infections among community members during routine surveillance of malaria. The problem is potentially higher in asymptomatic community members, who remain untreated but with high potential of sustaining transmission by carrying gametocytes which are the transmissible form of *Plasmodium* parasites. In areas with biological threats, such as artemisinin partial resistance (ART-R), it is critical to deploy and use highly sensitive tests as part of the response strategy to prevent the spread of resistant parasites. This study aimed to determine the performance of RDTs and microscopy using qPCR as a reference method for the detection of *Plasmodium* parasites in community members (with or without symptoms) in Kyerwa district of Kagera region, an area where ART-R has been recently confirmed [9]. The findings of this study provide evidence for the potential use of RDTs and microscopy in the surveillance and targeting of community infections (mainly asymptomatic individuals) as part of the response to ART-R in Tanzania.

## Methods

### Study design and sites

**Fig. 1:**
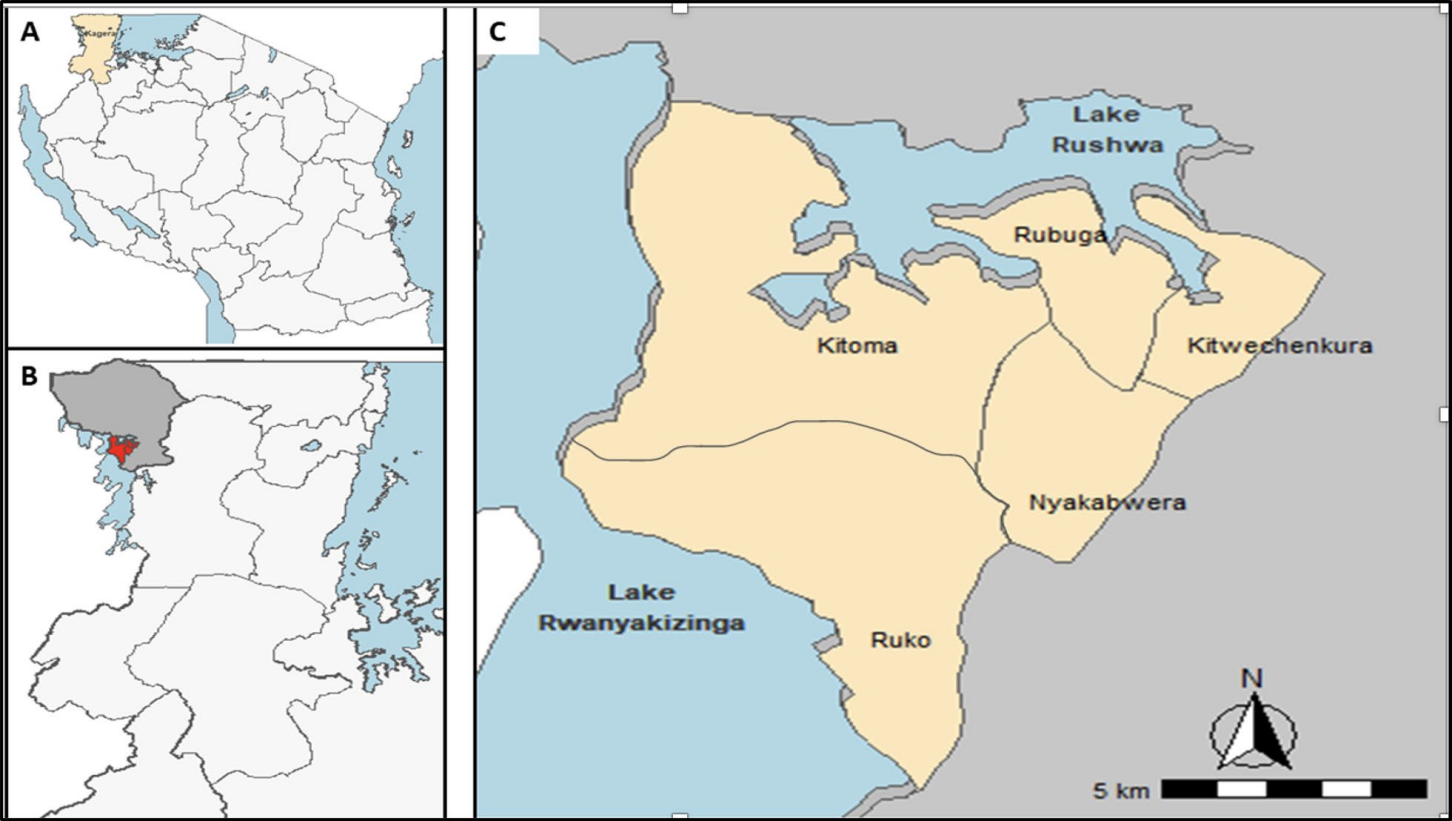
**A** Map of Tanzania showing the 26 regions including Kagera (gold), **B** study area in Kyerwa district (red), and (**C**) study villages (gold)

The data and samples used in this study were obtained from a community cross-sectional survey (CSS) that was conducted during the peak of malaria transmission season in five villages in Kyerwa district of Kagera region as previously described [33]. The initial study and the current work which is a follow-up analysis of samples and metadata were conducted as part of the main project on molecular surveillance of malaria in Mainland Tanzania (MSMT) which has been implemented in regions with varying endemicity since 2021 [3, 4, 33]. The five study villages (Kitoma, Kitwechenkura, Nyakabwera, Rubuga and Ruko) are located in Kyerwa district, which is among the eight councils of Kagera region as previously described [33] (Fig. 1). The villages were selected based on recent research findings which showed that some areas of Kagera region have high levels of parasites with mutations associated with ART-R [9, 34].

### Study population, participant enrollment, and data collection

In this study, the data and samples used for analysis were collected during a CSS that recruited community members aged 6 months and above. The participants were residents of the five villages of Kyerwa district that are part of the longitudinal component of the MSMT project. All potential participants were invited and asked to provide informed consent, and only those who provided consent participated in the study. Details of the procedures for enrollment of participants in the CSS were fully described in a recently published paper [33]. Briefly, demographic, anthropometric, clinical, and parasitological data were collected using questionnaires which were developed and run on tablets installed with an Open Data Kit (ODK) software version 4.2 [33]. Participants were appropriately identified using their permanent identification numbers (IDs), interviewed to collect demographic and anthropological data, clinically assessed and examined for any illness, and tested for malaria using RDTs as described earlier [33].

### Sample size and sample collection

The CSS aimed to recruit and collect blood samples and data from selected individuals who were conveniently enrolled from a population of 17,519 residents, in 4144 households, as previously described [33]. Through a convenient and non-random sampling approach, 4454 (25.4%) individuals from 768 (18.5%) households were enrolled. Enrolled participants provided finger-prick blood samples for RDTs, thick and thin blood smears for detection of *Plasmodium* parasites by microscopy, and dried blood spots (DBS) on Whatman No. 3 filter papers (GE Healthcare Life Sciences, PA, USA) for laboratory analyses. All samples were collected following the standard operating procedures (SOPs) of the MSMT project. Briefly, each DBS had three spots, each with a diameter of about 20 mm which is equivalent to about 50 μl of blood [3]. DBS samples were air-dried and packed in zipper bags with silica gel to prevent moisture and fungal growth and were stored at room temperature before shipment to the Genomics Laboratory at the National Institute for Medical Research (NIMR) in Dar es Salaam for further processing and analyses. Thick and thin blood smears were prepared in the field, air-dried, and thin smears were fixed with absolute methanol. The slides were stained on the same or the following day with 5% Giemsa solution for 45 min, and packed in slide storage boxes [35].

### Malaria diagnosis using RDT

Detection of malaria infections was done using RDTs under field conditions in which finger-prick blood was collected from all enrolled participants. Two brands of RDTs were used, Abbott Bioline Malaria Ag Pf/Pan (Standard Diagnostics Inc., Suwon City, South Korea) and Smart Malaria Pf/Pan Ag Rapid Test (Zhejiang Orient Gene Biotech Co. Ltd, Huzhou, Zhejiang, China). Both RDTs detected PfHRP2 and pLDH antigens of *Plasmodium* parasites. The tests were performed by experienced laboratory technologists and the results were interpreted following the manufacturers’ instructions [36]. RDT results were sent to clinicians to make a final diagnosis in case malaria infections were suspected. Individuals with positive results but without a history of using anti-malarial drugs in the last seven days were treated according to the national guidelines for diagnosis and treatment of malaria [37].

### Detection of *Plasmodium* parasites by microscopy

The collected blood smears were read at the the National Institute for Medical Research (NIMR) Genomics Laboratory in Dar es Salaam after the completion of field activities. Two experts, WHO-certified microscopists read the blood smears for detection of malaria parasites, identification and quantitation of asexual and sexual (gametocytes) stages, and detection of different *Plasmodium* species as described earlier [22]. In case of discrepancy, a third reading was performed by an independent microscopist blinded to the results of the first two microscopists. In all positive smears, asexual and sexual parasites were counted against 200 and 500 white blood cells (WBCs), respectively. Parasite density was obtained by multiplying the parasite counts by 40 for asexual and 16 for sexual parasites, assuming each microliter of blood contained 8000 WBCs [38]. A blood slide was considered negative for *Plasmodium* species if no parasites were detected in at least 200 oil-immersion, high-power fields on the thick film. Quality control of blood smears was done as previously described [22].

### DNA extraction and detection of *Plasmodium* parasites using real-time qPCR

DNA was extracted from three punches of DBS, and each punch had 6 mm, representing approximately 25 μl of blood. The extraction was done using 0.5% Chelex-Tween 20 (Bio-Rad Laboratories, Hercules, CA, USA), and the final DNA was eluted in a volume of approximately 100 μl of nuclease-free water as previously described [3]. Species-specific qPCRs targeting 18S ribosomal Ribonucleic acid (rRNA) subunit were performed as described earlier [3], and the reactions were run on the Bio-Rad CFX 96 Opus real-time PCR detection system (Bio-Rad Laboratories, Hercules, CA, USA), with CFX Maestro software version 2.2. A separate qPCR assay was run for each species: *P. falciparum, P. malariae,* and *P. ovale* spp. (simultaneously detecting both *P. ovale curtis* and *P. ovale wallikeri*) in a final volume of 12.5 µL reaction mixture containing 10uL of master mix and 2.5uL of DNA template. Each qPCR reaction was run for 40 cycles for all species except *P. malariae* which was run for 45 cycles, and quantification of the parasites was done by running standard curves using a tenfold serial dilution of engineered plasmids as earlier described [3]. Analysis of *P. vivax* was not done because it was rarely detected in the recent studies undertaken by the current authors [3, 4], but this will be done in the future using a pooling strategy that is being optimized.

### Data management and analysis

All clinical and parasitological data were collected using electronic data capture tools installed on tablets using ODK software version 4.2. The data were transmitted to the central server at NIMR in Dar es Salaam in real-time or once the internet connection was working, and they were validated daily, concurrent with field activities. After completion of field activities, the data were downloaded into Microsoft Excel for further cleaning. Data analysis was done using STATA software version 13 (StataCorp, Texas, USA) and R Programming language v4.4.1(R Foundation for Statistical Computing, Vienna, Austria). Descriptive statistics including frequencies, percentages, medians, and interquartile ranges (IQRs) were used to summarize the data. Using qPCR as the gold standard, the sensitivity, specificity, positive and negative predictive values, and the accuracy of RDTs and microscopy were determined using 2 × 2 contingency tables as previously described [39, 40]. For the three diagnostic techniques (RDTs, microscopy, and qPCR), the Pearson Chi-squared test was used to assess the differences in the prevalence of *Plasmodium* parasite infections among individuals of different age groups (under-fives (aged < 5 years), school children (aged 5–< 15 years), and adults with ≥ 15 years old), sex, history of fever in the past 48 h, fever at presentation (axillary temperature ≥ 37.5 °C), and their area of residence. Parasite densities from microscopy and qPCR results were summarized as geometric mean using STATA software version 13 (StataCorp, Texas, USA). A t-test or ANOVA was used to evaluate the differences in geometric mean parasite densities across sex and age groups. Predictors and determinants of sensitivities of RDTs and microscopy were computed using a multivariate logistic regression model, with adjustments for sex, age groups, history of fever in the past 48 h, fever at presentation, parasite densities (< 100, 100–1000, 1001–5000, 5001–10000, and > 10,000 asexual parasites/µL), and area of residence. For predictors and determinants of the specificity of RDTs and microscopy, adjustments were done for sex, age groups, history of fever in the past 48 h, fever at presentation, and area of residence. All results with a p-value < 0.05 were considered significant. Concordance between the three methods was calculated using Cohen's Kappa coefficient (κ), and *κ* < 0.20 indicated poor agreement, 0.21–0.40 was considered to be fair, 0.41–0.60 was moderate, 0.61–0.80 was good agreement, 0.81–0.99 was very good and 1.00 indicated perfect agreement [16].

## Results

### Characteristics of the study population

The CSS collected data and samples from 4454 individuals in the five villages of Kyerwa district in Kagera region. The data and samples from all participants were available and were used in this study. The median age of study participants was 14.0 (IQR = 6.7–36.0) years; 59.3% were females and the rest were males (40.7%). Of all individuals, 48.2% were adults (aged ≥ 15 years; n = 2146), followed by school children (aged 5–< 15 years) who accounted for 33.1% (n = 1473) while under-fives (aged < 5 years) accounted for 18.7% (n = 835). Among the five villages, Nyakabwera had 27.9% (n = 1243) of the study participants and Rubuga had 21.9% (n = 974), while each of the three remaining villages had less than 20.0% but with above 15.0% of the participants (Kitwechenkura with 17.3% (n = 769); Ruko had 17.0% (n = 759); and Kitoma had 15.9% (n = 709)). Overall, 30.1% of the participants had a history of fever in the past 48 h before the survey, while only 3.1% had a fever at presentation (with axillary temperature ≥ 37.5 °C). Among all participants, 6.5% had a history of medication use in the past seven days, and 5.8% reported that they used artemether-lumefantrine (AL) for the treatment of uncomplicated malaria (Table 1). Other anti-malarial drugs reported to have been used included injectable artesunate (by 0.07%, n = 3), Metakelfin (by 0.04%, n = 2), Quinine (0.02%, n = 1), and other unspecified anti-malarial drugs (0.02%, n = 1).

**Table 1:** Characteristics of study participants

Characteristics	Kitoma	Kitwechenkura	Nyakabwera	Rubuga	Ruko	Total
Examined, n (%)	709 (15.9)	769 (17.3)	1243 (27.9)	974 (21.9)	759 (17.0)	4454
Sex, n (%)						
Female	393 (55.4)	459 (59.7)	749 (60.3)	584 (60.0)	458 (60.3)	2643 (59.3)
Male	316 (44.6)	310 (40.3)	494 (39.7)	390 (40.0)	301 (39.7)	1811 (40.7)
p-value	0.004	< 0.001	< 0.001	< 0.001	< 0.001	< 0.001
Age in years, median (IQR)	16 (8–38)	15 (5–37)	14 (6–35)	12 (5–33)	14(7–36)	14.1 (6.7–36.0)
Age group (years), n (%)						
< 5	154 (21.7)	131 (17.0)	205 (16.5)	180 (18.5)	165 (21.7)	835 (18.8)
5–< 15	205 (28.9)	230 (29.9)	413 (33.2)	351 (36.0)	274 (36.1)	1473 (33.1)
15 +	350 (49.4)	408 (53.1)	625 (50.3)	443 (45.5)	320 (42.2)	2146 (48.2)
p-value	< 0.001	< 0.001	< 0.001	< 0.001	< 0.001	< 0.001
History of fever in the past 48 h, n (%)						
Yes	296 (41.8)	163 (21.2)	130 (10.5)	436 (44.8)	316 (41.6)	1341 (30.1)
No	413 (58.3)	606 (78.8)	1113 (89.5)	538 (55.2)	443 (58.4)	3113 (69.9)
	0. < 0.001	< 0.001	< 0.001	0.001	< 0.001	< 0.001
Fever at presentation (temp ≥ 37.5 °C), n (%)						
Yes	30 (4.2)	21 (2.7)	23 (1.9)	41 (4.2)	21 (2.8)	136 (3.1)
No	679 (95.8)	748 (97.3)	1220 (98.1)	933 (95.8)	738 (97.2)	4318 (96.9)
p-value	< 0.001	< 0.001	< 0.001	< 0.001	< 0.001	< 0.001
History of use of any medication in the past seven days, n (%)						
Yes	60 (8.5)	54 (7.0)	34 (2.7)	64 (6.6)	77 (10.1)	289 (6.5)
No	649 (91.5)	715 (93.0)	1209 (97.3)	910 (93.4)	682 (89.9)	4195 (94.5)
p-value	< 0.001	< 0.001	< 0.001	< 0.001	< 0.001	< 0.001
History of AL use in the past seven days, n (%)						
Yes	55 (7.8)	46 (6.0)	29 (2.3)	62 (6.4)	66 (8.7)	258 (5.8)
No	654 (92.2)	723 (94.0)	1214 (97.7)	912 (93.6)	693 (91.3)	4196 (94.2)
p-value	< 0.001	< 0.001	< 0.001	< 0.001	< 0.001	< 0.001

n: Number of observations; temp: Axillary temperature (≥ 37.5 °C); IQR: Inter-quartile range; AL: Artemether-Lumefantrine

### Prevalence of infections caused by *Plasmodium* parasites detected using RDTs, microscopy, and qPCR

All enrolled participants (n = 4454) were tested and the results were generated for the three methods, 44.4% had positive results by RDTs, while 32.1% were detected by microscopy and 39.8% by qPCR. The differences in the prevalence of *Plasmodium* infections by the three methods were statistically significant (p < 0.001). The highest prevalence of *Plasmodium* parasites as detected by RDT (68.5%) and microscopy (51.6%) was in Ruko, while by qPCR, the highest prevalence was in Rubuga (55.9%); with significantly high variations among the villages (p < 0.001). Nyakabwera had the lowest prevalence by all methods; with 14.5% detected using RDTs, 9.3% by microscopy and 13.9% by qPCR; and the differences in the prevalence detected by the three methods were statistically significant (p < 0.001 for all tests) (Table 2).

**Table 2:** Prevalence of *Plasmodium* parasites detected using RDT, microscopy and qPCR

Variable	# Positive by RDT, (%)	# Positive by microscopy, (%)	#Positive by qPCR, (%)
Overall (n = 4454)	1979/4454 (44.4)	1431/4454 (32.1)	1771/4454 (39.8)
Sex, n (%)			
Female	1089/2643 (41.2)	808/2643 (30.6)	992/2643 (37.5)
Male	890/1811 (49.1)	623/1811 (34.4)	779/1811 (43.0)
p-value	< 0.001	0.007	< 0.001
Age group (years), n (%)			
< 5	452/835 (54.1)	259/835 (31.0)	335/835 (40.1)
5–< 15	874/1473 (59.3)	681/1473 (46.2)	794/1473 (53.9)
≥ 15	653/2146 (30.4)	491/ 2146 (22.9)	642/2146 (29.9)
p-value	< 0.001	< 0.001	< 0.001
Village, n (%)			
Kitoma	440/709 (62.1)	346/709 (48.8)	395/709 (55.7)
Kitwechenkura	241/769 (31.3)	176/769 (22.9)	259/769 (33.7)
Nyakabwera	180/1243 (14.5)	116/1243 (9.3)	173/1243 (13.9)
Rubuga	598/974 (61.4)	401/974 (41.2)	544/974 (55.9)
Ruko	520/759 (68.5)	392/759 (51.6)	400/759 (52.7)
p-value	< 0.001	< 0.001	< 0.001
History of fever in the past 48 h, n (%)			
Yes	1186/1341 (88.4)	846/1341 (63.1)	1021/1341 (76.1)
No	793/3113 (25.5)	584/3113 (18.8)	750/3113 (24.1)
p-value	< 0.001	< 0.001	< 0.001
Fever at presentation (axillary temp ≥ 37.5 °C), n (%)			
Yes	108/136 (79.4)	93/136 (68.4)	101/136 (74.3)
No	1871/4318 (43.3)	1338/4318 (31.0)	1670/4318 (38.7)
p-value	< 0.001	< 0.001	< 0.001
History of using AL in the past seven days, n (%)			
Yes	249/258 (96.5)	97/258 (37.6)	165/258 (64.0)
No	1730/4196 (41.2)	1334/4196 (31.8)	1606/4196 (38.3)
p-value	< 0.001	0.053	< 0.001

n: Number of observations; RDTs: Rapid diagnostic tests; qPCR: Quantitative Polymerase Chain Reaction; AL: Artemether-Lumefantrine; temp: Axillary temperature; #: number of individuals with positive results

**Fig. 2:**
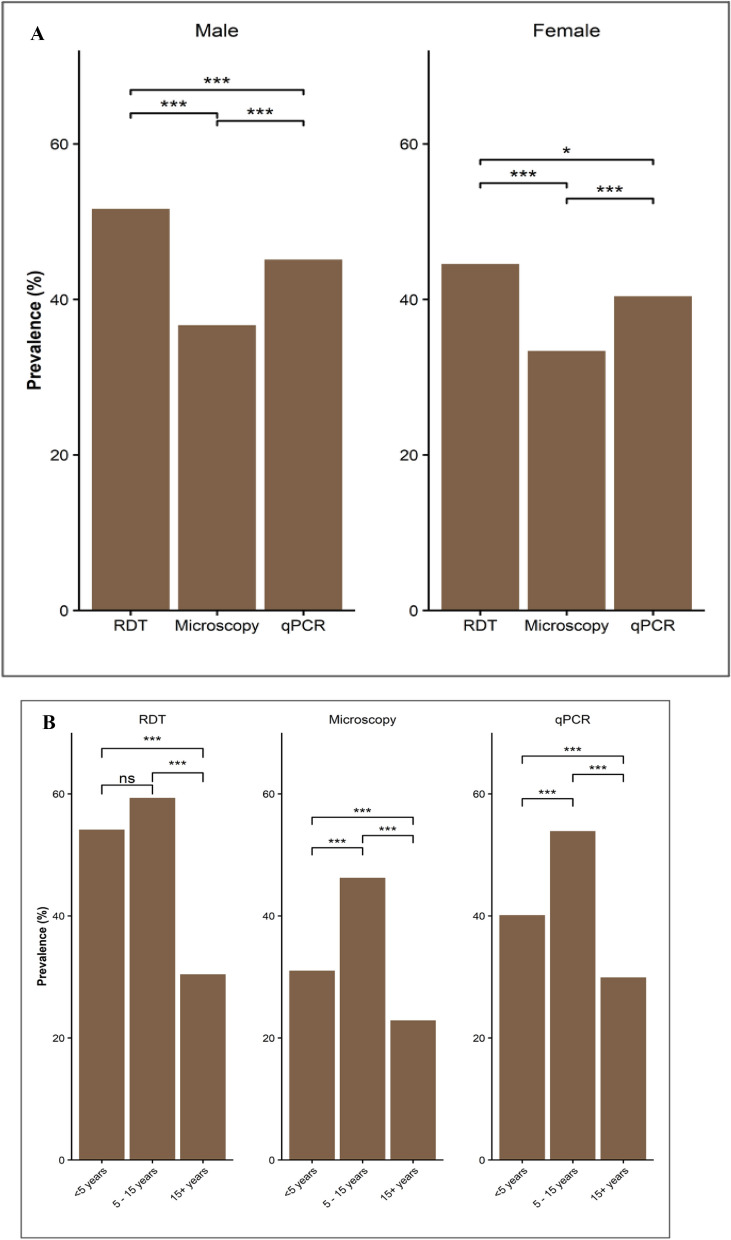
**B** Prevalence of infections caused by *Plasmodium* parasites detected using RDTs, microscopy, and qPCR among individuals of different age groups. ***p = 0.001, **p < 0.01, ns = Not significant. **A** Prevalence of infections caused by *Plasmodium* parasites detected using RDTs, microscopy, and qPCR among male and female participants. ***p < 0.001, **p < 0.01, *p < 0.05

Using all three methods, males (p ≤ 0.007) and school children (p < 0.001) had significantly higher prevalence of infections by *Plasmodium* species, but for RDTs the difference in the prevalence among under-fives and school children was not statistically significant (Table 2, Fig. 2A, B). Among individuals with a history of fever within the past 48 h, the majority tested positive by all methods (88.4% by RDTs; 63.2% by microscopy; and 76.1% by qPCR). The prevalence was also higher for those with fever at presentation (axillary temperature ≥ 37.5 °C), with 79.4% by RDTs, 68.4% by microscopy, and 74.3% by qPCR. Of the participants who reported that they used AL within the past seven days (n = 258), 96.5% had positive results by RDTs; 37.6% were positive by microscopy and 64.0% had positive results by qPCR (Table 2).

### Prevalence of *Plasmodium falciparum*, non-falciparum species and parasitaemia

Based on microscopy, the prevalence of *P. falciparum, P. malariae,* and *P. ovale* mono-infections in all samples (n = 4454) was 28.7%, 0.2%, and 0.3%, respectively. Mixed infections occurred in 3.0% of the samples, and these included double infections, with 2.1% of *P. falciparum/P. malariae* and 0.9% with *P. falciparum/P. ovale*, and triple infections of *P. falciparum/P. malariae/P. ovale* which occurred in one sample (0.02%)*.* By qPCR, the prevalence of *P. falciparum*, *P. malariae,* and *P. ovale* mono-infections in all samples (n = 4454) was 35.3%, 0.4% and 0.5%, respectively. The proportion of samples with positive results due to mixed infections of different *Plasmodium* species by qPCR was 3.6%. These mixed infections included double infections of *P. falciparum/P. malariae* at 0.9%*, P. falciparum/P. ovale* at 2.6%, and *0.04%* had *P. ovale/P. malariae,* while triple infections of *P. falciparum/P. malariae/P. ovale* occurred in 0.07% of the samples (Table 3)*.*

**Table 3:** Prevalence of *Plasmodium* species by microscopy and qPCR

*Plasmodium* species	Microscopy, n (%)	qPCR, n (%)
All species (n = 4454)	1431(32.1%)	1771(39.8%)
*P. falciparum*	1279 (28.7)	1566 (35.2)
*P. ovale*	12 (0.3)	24 (0.5)
*P. malariae*	7 (0.2)	17 (0.4)
*P. falciparum/P. malariae*	93 (2.1)	40 (0.9)
*P. falciparum/P. ovale*	39 (0.9)	119 (2.7)
*P. ovale/P. malariae*	0 (0)	2 (0.04)
*P. falciparum/P. malariae/P. ovale*	1 (0.02)	3 (0.07)

n: Number of observations; qPCR: Quantitative Polymerase Chain Reaction

**Fig. 3:**
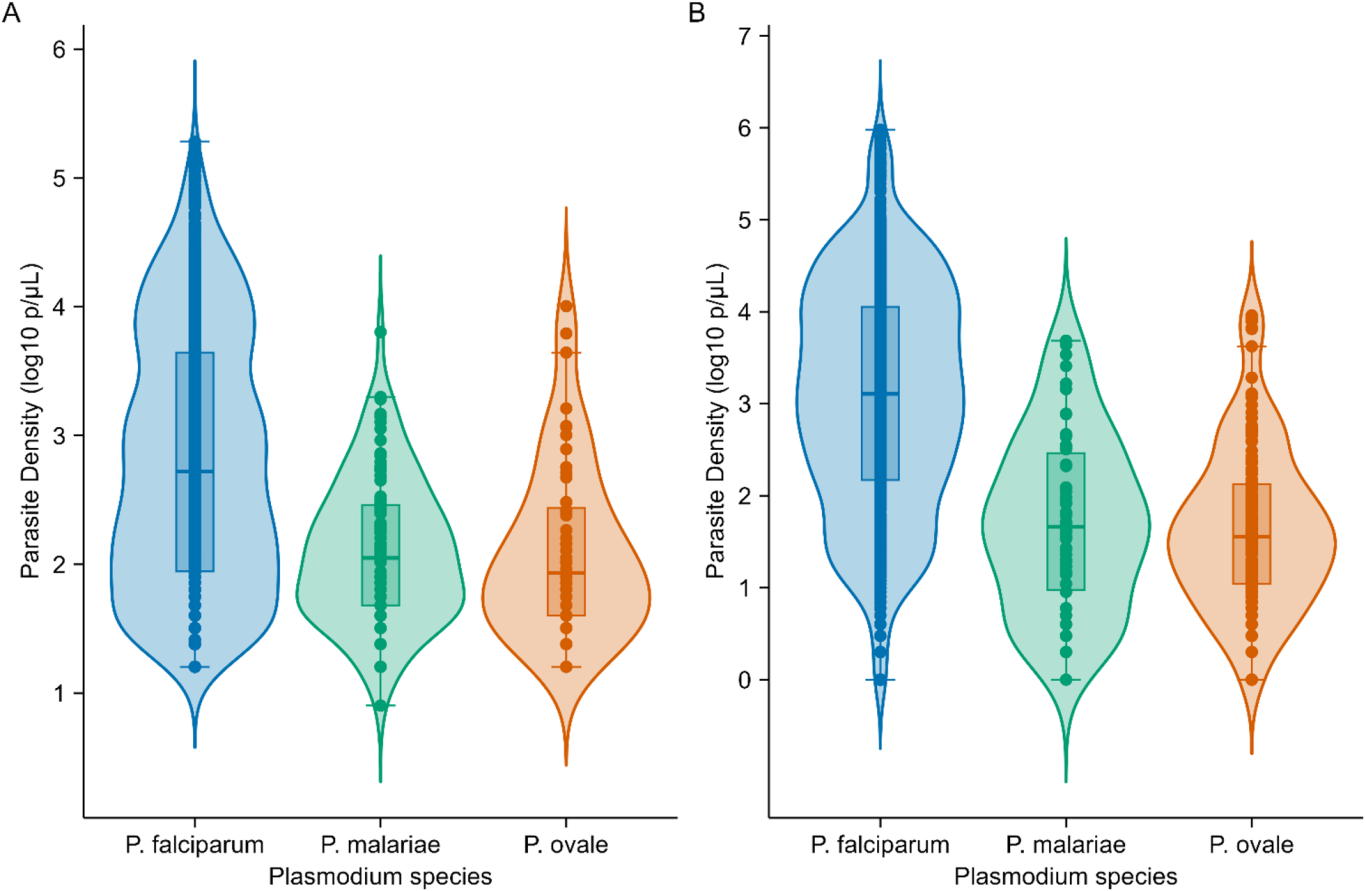
Violin plots representing the distribution of parasite densities of *P. falciparum, P. malariae,* and *P. ovale*. **A** shows densities detected using microscopy, while panel (**B**) presents the densities detected using qPCR. The plots highlight the variability in parasite densities for each species, with the shape and width of the violins indicating the spread and frequency of the data points

The geometric mean parasite densities (GMPDs) by microscopy were 642 parasites/µL (95% CI = 570–723) for *P. falciparum,* 124 parasites/µL (95% CI = 97–160) for *P. malariae*, and 126 parasites/µL (95% CI = 82–194) for *P. ovale* (Fig. 3A). The parasite densities of *P. falciparum* were significantly lower (p < 0.001) among adults aged ≥ 15 years while the highest densities were among under-fives, with 1915 parasites/µL (95% CI = 1398–2623) (Table 4). The GMPDs of *P. falciparum* were higher in males compared to females (p = 0.014) (Table 4). By qPCR, the GMPDs of *P. falciparum, P. ovale,* and *P. malariae* were 1180 parasites/µl (95% CI = 1032–1349), 44 parasites/µl (95% CI = 32–61), and 50 parasites/µL (95%CI = 29–89) (Fig. 3B). The parasite densities of *P. falciparum* were lower among adults aged ≥ 15 years, compared to under-fives who had higher densities (p < 0.001). The mean parasite densities for *P. ovale* were also significantly higher among under-fives (p = 0.002), while the GMPD of *P. malariae* in under-fives was also higher, but the differences among age groups were not statistically significant (p = 0.272). Based on qPCR, the parasitemia due to *P. falciparum* was higher in males (p = 0.015) while with *P. malarie* and *P. ovale,* the differences in GMPD among females and males were not statistically significant (p = 0.528, and p = 0.225 for *P. malarie*, and *P. ovale*, respectively) (Table 4).

**Table 4:** Geometric mean parasite density (GMPD) of *Plasmodium* parasites detected by microscopy and qPCR

Variable	*Plasmodium* species GMPD (95% CI) by microscopy (n = 1431)			*Plasmodium* species GMPD (95% CI) by qPCR (n = 1771)		
	*P. falciparum (n* = *1412)*	*P. ovale (n* = *52)*	*P. malariae (n* = *101)*	*P. falciparum (n* = *1728)*	*P. ovale (n* = *148)*	*P. malariae (n* = *62)*
Overall GMPD (95%CI)	642 (570–722)	126 (82–194)	124 (97–160)	1180 (1032–1349)	44 (32–61)	50 (29–86)
Sex						
Male	759 (635–908)	118 (67–209)	114 (79–163)	1401 (1143–1717)	37 (24–57)	44 (17–116)
Female	563 (481–660)	136 (67–276)	136 (95–195)	1017 (849–1219)	50 (30–85)	37 (16–85)
p-value	0.014	0.747	0.477	0.015	0.225	0.528
Age group						
< 5 years	1915 (1398–2623)	209 (70–629)	288 (165–504)	2076 (1462–2948)	118 (59–236))	67 (15–300)
5–< 15 years	850 (724–998)	94 (54–164)	105 (80–137)	1679 (1386–2034)	36 (23–56)	46 (23–91)
≥ 15 years	246 (206–293)	166 (57–481)	63 (28–142)	566 (460–697)	21 (10–42)	25 (7–91)
p-value	< 0.001	0.458	< 0.001	< 0.001	0.002	0.272

GMPD: Geometric Mean Parasite Density; n = Sample size

Sample size for each of the *Plasmodium* species included mixed infection as categorized in Table 3

**Fig. 4:**
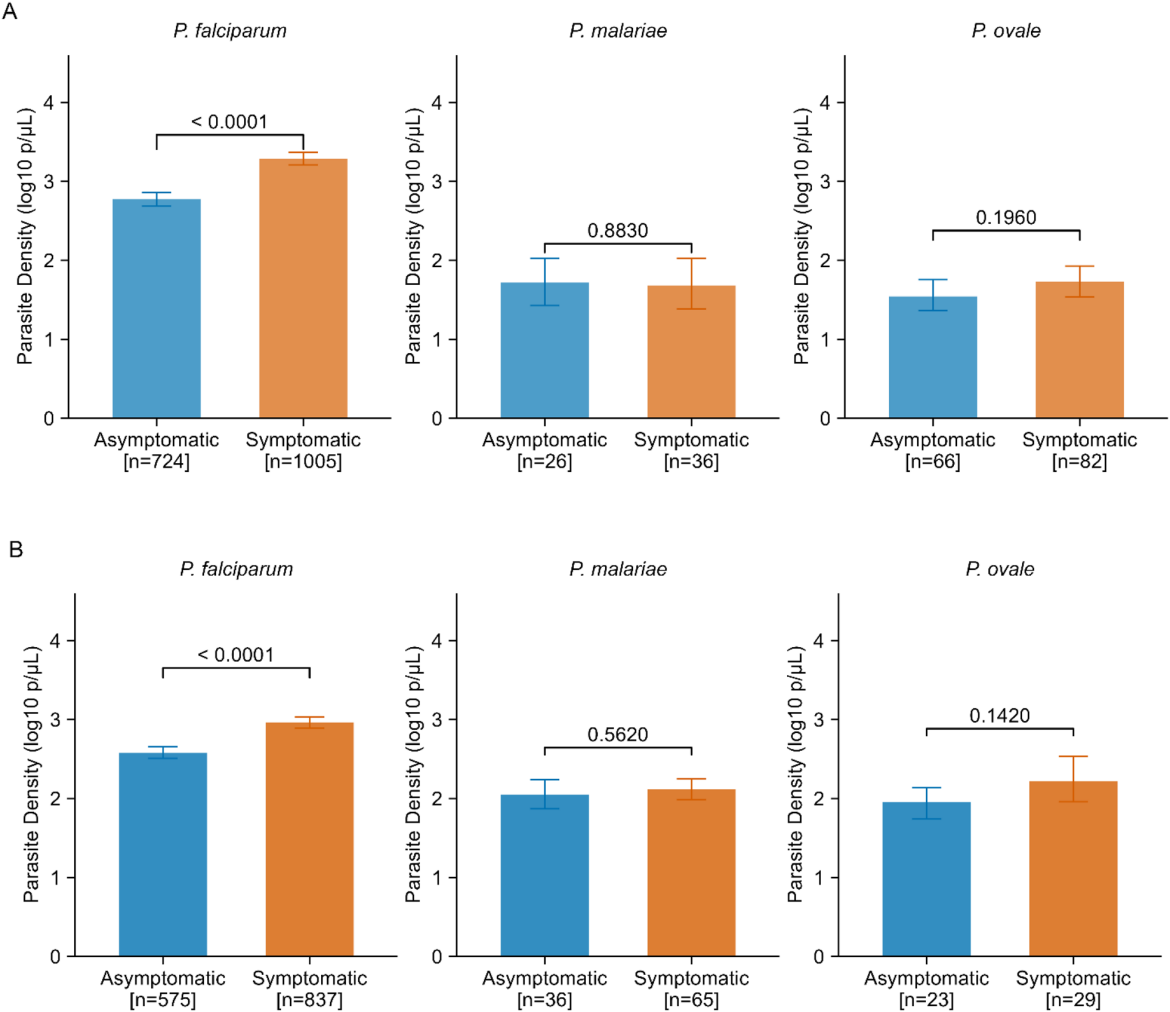
Bar graphs showing the geometric mean parasite densities of different *Plasmodium* species among symptomatic (brown bars) and asymptomatic (blue bars) individuals as detected by qPCR (**A**) and microscopy (**B**). Error bars represent 95% confidence intervals

The GMPDs of *P. falciparum* by qPCR and microscopy were significantly higher (p < 0.001) among symptomatic individuals with GMPD of 1938 parasites/µL (95% CI = 233–16,130) by qPCR and 921 parasites/µL (95% CI = 114–7460 by microscopy; compared to asymptomatic individuals (with GMPD of 593 parasites/µL, 95% CI = 75.7–4644 by qPCR and 379 parasites/µL, 95% CI = 49.6–2898 by microscopy). In contrast, GMPDs of *P. malariae* did not differ significantly among  symptomatic and asymptomatic individuals, with GMPD of 48.1 parasites/µL (95% CI 5.95–390) by qPCR and 112 parasites/µL (95% CI 15.1–836) by microscopy for symptomatic individuals, and 52.2 parasites/µL (95% CI 6.85–398) by qPCR and 131 parasites/µL (95% CI 17.3–1000) by microscopy for asymptomatic individuals (p = 0.883 for qPCR and p = 0.562 for microscopy) (Fig. 4). However, the GMPDs of P. *ovale* spp. were slightly higher in symptomatic individuals (53.6 parasites/µL, 95% CI 6.71–427 by qPCR and 165 parasites/µL, 95% CI 21–1296 by microscopy) compared to asymptomatic individuals (34.8 parasites/µL, 95% CI 4.58–265 by qPCR and 89.8 parasites/µL, 95% CI 12.1–664 by microscopy), but the differences were not statistically significant (p = 0.196 for qPCR and p = 0.142 for microscopy) (Fig. 4).

### Performance of RDTs, microscopy, and qPCR for detection of malaria parasites

**Fig. 5:**
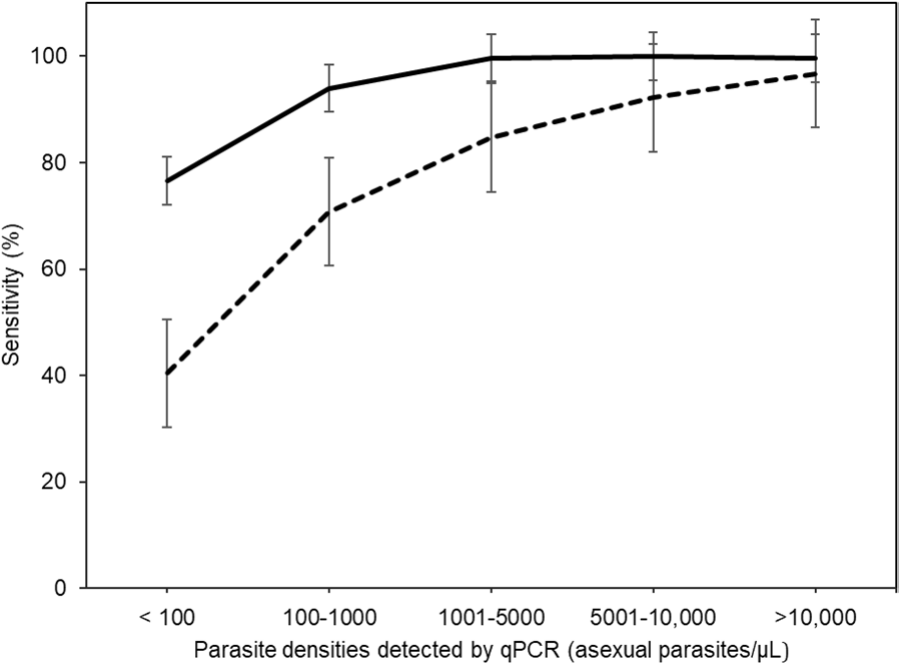
Sensitivity of RDTs (solid line) and microscopy (dotted line) at different levels of parasitemia, as determined using qPCR

Of all participants (n = 4454), 1651 (37.1%, 95% CI = 35.3–38.9) were positive by both RDTs and qPCR, while 1328 participants (29.8%, 95% CI = 28.2–31.5) tested positive by both microscopy and qPCR (Table 5). With qPCR as the reference method, the sensitivity of RDTs and microscopy was 93.2% (95% CI = 92.0–94.4) and 75.0% (95% CI = 72.9–77.0), respectively. The specificity of RDTs was 87.8% (95% CI = 86.5–89.0), while that of microscopy was 96.2% (95% CI = 95.4–96.9) (Table 5 and Fig. 5). The diagnostic accuracy of RDTs was 89.9% (95% CI = 89.0–90.8), and the accuracy of microscopy was 87.7% (86.7–88.7). Microscopy showed a higher positive predictive value of 92.8% (95% CI = 91.4–94.0) compared to RDTs which had a positive predictive value of 83.4% (95% CI = 82.0–84.8). Conversely, the negative predictive value was higher for RDT than for microscopy, 95.2% (95% CI = 94.3–95.9) and 85.30% (95% CI = 84.3–86.3), respectively. Both RDTs and microscopy had better agreement with qPCR, with Cohen’s kappa values of 0.79 (95% CI = 0.78–0.81) and 0.74 (95% CI = 0.71–0.76), respectively (Table 5).

**Table 5:** Contingency and Diagnostic Performance of RDT and Microscopy using qPCR as the reference method

	qPCR			Value (95% Confidence interval)					
	Positive	Negative	Total	Sensitivity	Specificity	PPV	NPV	Accuracy	*K*-value
RDT									
Positive	1651 (83.4%)	328 (12.2%)	1979 (44.4%)	93.2% (92.0–94.4)	87.8% (86.5–89.0)	83.4% (82.0–84.8)	95.2% (94.3–95.9)	89.9% (89.0–90.8)	0.79 (0.78–0.81)
Negative	120 (6.8%)	2355 (95.2%)	2475 (55.6%)						
Total	1771	2683	4454						
Microscopy									
Positive	1328 (92.8%)	103 (3.8%)	1,431 (32.1%)	75.0% (72.9–77.0)	96.2% (95.4–96.9)	92.8% (91.4–94.0)	85.3% (84.3–86.3)	87.7% (86.7–88.7)	0.74 (0.71–0.76)
Negative	443 (25.0%)	2580(85.3%)	3023 (67.9%)						
Total	1771	2683	4454						

qPCR: quantitative Polymerase chain reaction, PPV: Positive predictive value, NPV: Negative predictive value, K-value: Cohen's Kappa value

The sensitivity of RDTs and microscopy varied across different levels of parasitemia when compared to qPCR-detected parasite densities. Microscopy had the lowest sensitivity (40.4%) at < 100 parasites/μL, while the lowest sensitivity of RDTs at parasitaemia < 100 parasites/μL was 76.6% (Fig. 5). The sensitivity of both RDTs and microscopy increased with an increase in parasitemia, from 70.7% and 94.0% at 100–1000 parasites/μL to 92.3% and 100% at > 5000–10000 parasites/μL by microscopy and RDTs, respectively. At very high parasite densities (> 10,000 parasites/μL), both RDTs and microscopy demonstrated a sensitivity above 99.6% as shown in Fig. 5. The sensitivity of both RDTs and microscopy was significantly higher at high parasitaemia (aOR > 100.0, p < 0.001) and among individuals with a history of fever (aOR > 1.30, p < 0.001) (Table 6). The specificity of RDTs was significantly influenced by age (aOR > 0.40, p < 0.001), history of fever (aOR = 0.08, p < 0.001), and fever at presentation (aOR = 0.59, p < 0.05) while the specificity of microscopy remained unaffected by demographic and clinical variables (p > 0.05) (Table 7).

**Table 6:** Predictors and determinants of sensitivity of RDT and microscopy among individuals with positive results by qPCR

Variable/covariate	RDTs			Microscopy		
	Sensitivity (%, 95% CI)	Unadjusted OR (95% CI)	Adjusted OR (95% CI)	Sensitivity (%, 95% CI)	Unadjusted OR (95% CI)	Adjusted OR (95% CI)
Sex						
Male	745/779 (95.6, 94.0–96.9)	Ref	Ref	588/779 (75.5, 72.3–78.4)	Ref	Ref
Female	906/992 (91.3, 89.4–92.9	0.48 (0.32–0.72) ***	0.52 (0.31–0.86) *	740/992 (74.6, 71.8–77.2)	0.95 (0.77–1.18)	1.14(0.86–1.51)
Age group						
< 5 years	329/335(98.2, 96.1–99.2)	Ref	Ref	245/335(73.1, 68.1–77.6)	Ref	Ref
5–< 15 years	768/794(96.7, 95.2–97.8)	0.54 (0.20–1.24)	0.53 (0.18–1.34)	651/794 (82.0, 79.2–84.5)	1.67(1.23–2.26) ***	2.37 (1.58–3.55) ***
≥ 15 years	554/642 (86.3, 83.4–88.7)	0.11 (0.04–0.24) ***	0.18 (0.1–0.42) ***	432/642 (67.3, 63.6–70.8)	0.76 (0.56–1.01)	1.45 (0.98–2.15)
History of fever						
No	642/750 (85.6, 82.9–87.9)	Ref	Ref	507/750 (67.6, 64.2–70.9)	Ref	Ref
Yes	1009/1021 (98.8, 98.0–99.3)	14.14 (8.05–27.27) ***	8.78 (4.73–17.69) ***	821/1021 (80.4, 77.9–82.7)	1.97 (1.58–2.45) ***	1.35 (1.02–1.79) *
Fever at presentation						
No	1550/1670(92.8, 91.5–94.0)	Ref	Ref	1236/1670 (74.0, 71.976.1)	Ref	Ref
Yes	101/101 (100, 96.3–100)	1.29 (1.03–1.62) *	1.03 (0.76–1.39)	92/101 (91.1, 83.9–95.2)	1.24 (1.09–1.42) **	1.05 (0.88–1.26)
Parasite density						
< 100	302/391 (77.2, 72.8–81.1)	Ref	Ref	158/391 (40.4, 35.7–45.3)	Ref	Ref
100–1000	426/454 (93.8, 91.2–95.7)	1.39 (0.85–2.24)	1.75 (0.98–3.14)	321/454 (70.7, 66.4–74.7)	2.45(1.60–3.79) ***	2.81 (1.74–4.58) ***
1001–5000	338/339 (99.7, 98.3–99.9)	4.00 (2.34–6.99) ***	5.36 (2.82–10.48) ***	287/339 (84.7, 80.4–88.1)	5.11(3.37–7.85) ***	5.86 (3.66–9.52) ***
5001–10000	129/129 (100, 97.1–100)	44.21 (18.76–129.96) ***	56.76 (21.76–180.9) ***	119/129 (92.2, 86.3–95.7)	14.11 (9.45–21.38) ***	20.68 (13.0–33.61) ***
> 10,000	456/458 (99.6, 98.4–99.9)	81.51 (24.63–504.50) ***	104.67 (27.83–6927) ***	443/458 (96.7, 94.7–98.0)	70.92 (39.71–134.23) ***	146.1 (76.5–294.4) ***
Village of residence						
Kitwechenkura	220/259(84.9, 80.1–88.8)	Ref	Ref	167/259 (64.5, 58.5–70.1)	Ref	Ref
Nyakabwera	154/173(89.0, 83.5–92.9)	1.44 (0.81–2.63)	6.30 (2.90–14.2) ***	92/173 (53.2, 45.8–60.5)	0.63 (0.42–0.93) *	1.95 (1.16–3.28) *
Rubuga	361/395(91.4, 88.2–93.7)	1.88 (1.15–3.08) *	4.17 (2.12–8.41) ***	336/395 (85.1, 81.2–88.2)	3.14 (2.16–4.59) ***	9.27 (5.77–15.08) ***
Kitoma	529/544(97.2, 95.5–98.3)	6.25 (3.45–11.92) ***	9.49 (4.47–21.2) ***	374/544 (68.8, 64.7–72.5)	1.21 (0.89–1.65)	1.65 (1.11–2.46) *
Ruko	387/400(96.8, 94.5–98.1)	5.28 (2.83–10.47) ***	14.6 (6.55–34.6) ***	359/400 (89.8, 86.4–92.4)	4.82 (3.22–7.34) ***	14.8 (8.98–24.92) ***

OR Odds ratio, CI Confidence interval

***p < 0.001, **p < 0.01, *p < 0.05

**Table 7:** Predictors and determinants of specificity of RDT and microscopy among individuals with negative results by qPCR

Variable/covariate	RDTs			Microscopy		
	Specificity (%, 95% CI)	Unadjusted OR (95% CI)	Adjusted OR (95% CI)	Specificity (%, 95% CI)	Unadjusted OR (95% CI)	Adjusted OR (95% CI)
Sex						
Male	887/1032 (85.9, 83.7–87.9)	Ref	Ref	997/1032 (96.6, 95.3–97.6)	Ref	Ref
Female	1468/1651 (88.9, 87.3–90.3)	1.31 (1.04–1.65) *	1.01 (0.74–1.36)	1583/1651 (95.9, 94.8–96.7)	0.82 (0.53–1.23)	0.84 (0.54–1.28)
Age group						
< 5 years	377/500 (75.4, 71.4–79.0)	Ref	Ref	486/500 (97.2, 95.4–98.3)	Ref	Ref
5–< 15 years	573/679 (84.4, 81.5–86.9)	1.76 (1.32–2.36) ***	0.87 (0.59–1.28)	649/679 (95.6, 93.8–96.9)	0.62 (0.32–1.17)	0.47 (0.23–0.89) *
≥ 15 years	1405/1504 (93.4, 92.1–94.6)	4.63 (3.47–6.19) ***	4.69 (3.24–6.81) ***	1445/1504 (96.1, 95.0–96.9)	0.71(0.38–1.24)	0.62 (0.32–1.11)
History of fever						
No	2212/2363 (93.6, 92.6–94.5)	Ref	Ref	2286/2363 (96.7, 95.9–97.4)	Ref	Ref
Yes	143/320 (44.7, 39.3–50.2)	0.05 (0.04–0.07) ***	0.08 (0.06–0.12) ***	294/320(91.9, 88.4–94.4)	0.38 (0.24–0.61) ***	0.52 (0.32–0.87) *
Fever at presentation						
No	2327/2648 (87.9, 86.6–89.1)	Ref	Ref	2546/2648 (96.1, 95.3–96.8)	Ref	Ref
Yes	28/35 (80.0, 64.1–90.0)	0.55 (0.25–1.38)	0.59 (0.22–1.82)	34/35 (97.1, 85.5–99.5)	0.97 (0.74–1.26)	0.94 (0.72–1.23)
Village of residence						
Kitwechenkura	489/510 (95.9, 93.8–97.3)	Ref	Ref	501/510 (98.2, 96.7–99.1)	Ref	Ref
Nyakabwera	1044/1070 (97.6, 96.5–98.3)	1.72 (0.95–3.09)	1.96 (1.05–3.60) *	1046/1070 (97.8, 96.7–98.5)	0.78 (0.34–1.64)	0.80 (0.35–1.68)
Rubuga	235/314 (74.8, 69.8–79.3)	0.13 (0.08–0.21) ***	0.13(0.07–0.23) ***	304/314 (96.8, 94.2–98.3)	0.55 (0.21–1.37)	0.59 (0.23–1.49)
Kitoma	361/430 (84.0, 80.2–87.1)	0.22 (0.13–0.37) ***	0.32(0.18–0.55) ***	403/430 (93.7, 91.0–95.6)	0.27 (0.12–0.56) ***	0.29 (0.13–0.61) **
Ruko	226/359 (63.0, 57.8–67.8)	0.07 (0.04–0.12) ***	0.08 (0.05–0.14) ***	326/359 (90.8, 87.4–93.4)	0.18 (0.08–0.36) ***	0.20 (0.09–0.41) ***

OR Odds ratio, CI Confidence interval

***p < 0.001, **p < 0.01, *p < 0.05

## Discussion

This study utilized samples and data from a community CSS that was conducted in Kyerwa district as previously described [33], to assess the performance of three malaria diagnostic methods (RDTs, microscopy and qPCR) among community members (with or without symptoms of malaria). It focused on the detection of infections caused by different *Plasmodium* parasites among participants from the study communities, whereby the majority were asymptomatic, and some were symptomatic for malaria but had not visited the health facilities to seek health care. The study aimed to ascertain the performance of these tests for routine diagnosis and surveillance of malaria in areas where increasing levels of parasites with ART-R have been recently reported [34]. The study area is located near Rwanda and Uganda borders where there is high rate of human migration across the borders and ART-R has been confirmed in these countries based on WHO-recommended methods [9]. Thus, this area is of high interest, and it is being targeted as part of the response to ART-R with a broader focus on the Great Lakes Region of Africa. In this and other communities, recent studies have reported a high prevalence of *Plasmodium* species among asymptomatic individuals and identified vulnerable groups (eg. males, school children, individuals with low socio-economic status and those living in poorly constructed houses) which need to be targeted by malaria interventions including intensified surveillance based on the use of sensitive diagnostic methods [33, 41].

In this study, the overall prevalence of infections caused by *Plasmodium* species detected using RDTs was higher compared to the prevalence reported when parasites were detected with microscopy, and the prevalence was also higher among males, school children and individuals with a fever history or fever at presentation. The prevalence of infections caused by *Plasmodium* species when detection of parasites was done by RDTs was higher followed by detection using qPCR while parasite detection by microscopy had the lowest prevalence. The GMPD of *P. falciparum* was higher by qPCR compared to microscopy. Using qPCR as the gold standard, RDTs had a higher sensitivity compared to microscopy while the specificity was higher for microscopy compared to RDTs, and the sensitivity of both RDTs and microscopy increased with increasing parasite density. These findings support the use of RDTs as a reliable method for the detection of parasite antigens to support the targeting of community members, particularly asymptomatic individuals, and can potentially be utilized in the surveillance of malaria and the ongoing plans to develop a response to ART-R [9, 34].

The high overall prevalence of infections caused by *Plasmodium* species when detection was done by RDTs and even among individuals of different age groups and sex could be due to the persistence of HRP2 antigens, which can remain in the blood and be detectable for up to four weeks after effective treatment, and even after complete clearance of malaria parasites [34]. The higher prevalence of infections by *Plasmodium* species when parasite detection was done using RDTs could also be caused by human errors during the interpretation of results as some bands on test lines can be reported as present while the test is actually negative [42]. This means that some of the tests could actually be false RDTs positive, resulting in high false positive results which have been associated with unwarranted prescription of antimalarials. History of anti-malarial use within one to two weeks prior to testing may provisionally help to identify individuals with false positive results as shown in this study that such individuals were more likely to be positive by RDTs and qPCR compared to microscopy, but cannot rule out failed clearance (recrudescence) or new infection. These findings are similar to the studies conducted elsewhere, which reported a higher sensitivity of RDTs than microscopy [16, 43]. In contrast, some studies have reported that RDTs exhibit a lower sensitivity compared to microscopy in certain settings [44, 45]. These discrepancies highlight the importance of considering regional variations, parasite species distribution, and the performance characteristics of RDTs when interpreting diagnostic results.

The lower prevalence of infections caused by *Plasmodium* species as detected by microscopy compared to RDTs could be due to its low sensitivity, particularly among individuals with low parasite densities, particularly in asymptomatic individuals. The differences could also be due to the quality of blood smears, and other technical limitations of microscopy such as high detection limits when the reading of blood smears is done by microscopists with limited skills or experience. Previous studies have shown that low parasitaemia below the limit of detection of microscopy is associated with an increasing rate of false negative results since a number of positive samples with low-density infections are missed [46, 47]. Quality and technical limitations including the optical condition of microscopes, skills of microscopists, smearing and slide staining quality have also been reported to affect microscopy results [48–50]. With poorly prepared smears or faulty microscopes, even appropriately-trained readers can potentially miss or misdiagnose malaria. Poorly trained microscopists can also contribute to this problem even if the smears were well-prepared [48]. However, the study team used high-quality reagents and experienced experts suggesting that these factors could not have potentially affected the results of this study. Thus, more studies will be needed to further tease out the poor performance of microscopy in similar study groups and areas of comparable transmission intensities.

Parasite densities of *P. falciparum* detected by qPCR were significantly higher compared to those detected by microscopy while the GMPD of *P. malariae* and *P. ovale* were higher for microscopy compared to qPCR. Due to the low detection limit of qPCR which enables it to detect more samples especially those with low parasitaemia, the parasite densities of *P. falciparum* detected by qPCR were expected to be lower compared to those detected by microscopy. The reasons for high parasitaemia detected by qPCR compared to microscopy are not clearly known and will be explored in future studies which are planned in the same area and others under the MSMT project. Studies have shown that qPCR can identify as few as one parasite per microliter of blood, whereas microscopy typically requires a minimum threshold of around 50–100 parasites/μL to ensure accurate identification [16, 17]. Furthermore, parasitaemia due to *P. falciparum* was higher compared to *P. ovale* and *P. malariae*. This could be due to differences in the biology of these malaria parasite species whereby *P. falciparum* is known to be highly pathogenic compared to *P. ovale* and *P. malariae*, as it is capable of rapid replication and has the potential to evade the immune system leading to high parasitaemia [4, 51]. The higher parasitaemia of *P. falciparum* in under-fives could be due to a low level of immunity among under-fives, and this should always be considered when implementing case management strategies in under-fives [52]. The findings of this study are similar to what was reported in previous studies [53]. Moreover, parasitaemia due to both *P. falciparum* detected by qPCR and microscopy was significantly higher among symptomatic individuals compared to those who were asymptomatic. For *P. malariae* and *P. ovale,* the parasite densities were similar among symptomatic and asymptomatic individuals. The differences could probably be due to differences in the biology of these species and their ability to elicit immune responses to malaria infections [54].

While both RDTs and microscopy worked well, RDTs had a higher diagnostic accuracy than microscopy, and higher sensitivity but substantially lower specificity and PPV compared to microscopy. The specificity and PPV of RDTs were lower as expected, and it was most likely due to HRP2/3 protein residues that tend to persist even after the infection has been cleared with anti-malarials [30, 36]. RDTs were more sensitive than microscopy even at low parasitemia (< 100 asexual parasites/μL), with the sensitivity of the two methods increasing as parasite density increased. The lower sensitivity of microscopy was potentially attributed to lower parasite density, as this study focused on community members whereby majority of the study participants were asymptomatic, with some of them carrying low-density infections below the detection limit of microscopy. The false positivity rate was higher with RDTs (12.2%) compared to microscopy (3.8%) suggesting that using RDTs particularly in community members made of mainly asymptomatic individuals should be properly assessed to avoid unwanted prescription of antimalarials, while the false negativity rate was higher with microscopy (25.0%) and lower with RDTs (6.8%). This agrees with the historical use of microscopy as a confirmatory test for malaria diagnosis due to its higher specificity [55]. However, as demonstrated from this study, RDTs, which are the current option for malaria diagnosis at health facilities, had high sensitivity but low specificity, and microscopy which is the current gold standard, had high specificity but relatively lower sensitivity. Molecular detection by qPCR is highly sensitive and specific, but not feasible in clinical settings. More efforts need to be invested in research and development, to determine the most ideal approach for malaria diagnosis in areas with heterogeneous malaria transmission. Such tests will also be critical for supporting malaria surveillance within the ongoing elimination efforts and in responding to ART-R.

Parasite density, age, and sex were found to affect the sensitivity and specificity of RDTs and microscopy in various ways in this study. The sensitivity of both RDTs and microscopy increased with increasing parasite density and more false negatives were associated with low parasite densities. Similar to what has been reported by others [46, 56], the sensitivity of microscopy and RDTs becomes very low below 100 parasites/µL or < 0.002% parasitaemia for RDTs and < 50 parasites/µL or < 0.001% parasitaemia for microscopy. This implies that at low parasite densities, a considerable proportion of positive individuals may be missed by these tests, and this is of concern, especially in areas targeting elimination as individuals residing in these areas tend to have infections with low levels of parasitaemia. The sensitivity of RDTs decreased with increasing age and this was similar to what was reported by others [43, 57, 58]. This could be explained by age-dependent immunity which develops following repeated exposure to infections, that may suppress parasitaemia and result in low densities below the detection threshold [43, 59]. The specificity of RDTs increased with age, which is in agreement with what was previously reported by others [60–62], although other studies reported no age-specific trends [16, 63]. The effect of age on specificity is thought to be influenced by the parasite density, which is related to the improvement of the immune system with age [60].

This study had two limitations. Firstly, a history of fever within the past two days and a history of anti-malarial use within the previous seven days were based on self-reported information, increasing the potential of recall bias. Participants or guardians of participants were the only source of information, and the team had no means to ascertain their responses. However, the findings reported in this study are similar to what have been previously reported [22], suggesting that the responses reported in this study could potentially represent the actual status of fevers and use of anti-malarial drugs in the communities. Secondly, the study covered only one district of Kagera region where ART-R has recently been confirmed, and the Ministry of Health is planning to implement a response strategy for ART-R. Thus, the findings from this study cannot be used to represent general performance of these three diagnostic methods in other areas of Mainland Tanzania. Despite these limitations, the finding of this study demonstrates higher performance of RDTs compared to microscopy, with qPCR as the reference method, suggesting that RDTs can be used as reliable methods for detection of malaria in communities, with a focus on areas with reported ART-R or ongoing malaria elimination strategies.

## Conclusion

This study revealed that RDTs were more sensitive and accurate but less specific compared to microscopy in detecting malaria parasites among community members, with a high proportion of asymptomatic individuals. The false positivity rate was higher with RDTs while the rate of false negative results was higher with microscopy. The performances of both RDTs and microscopy were poor at very low parasite density (< 100 parasite/μL) but increased with an increase in parasite density. The higher sensitivity and diagnostic accuracy of RDTs compared to microscopy support the routine use of RDTs for case management and surveillance of malaria through health facilities and in the communities (targeting symptomatic and asymptomatic individuals) for malaria control and elimination, and for responding to ART-R in this area with confirmed ART-R. Due to the lower performance of microscopy particularly among individuals with low parasite densities, RDT usage in routine malaria diagnostic services should be prioritized, however, microscopy should be utilized for malaria confirmation purposes due to its high specificity. To ensure the high quality of malaria diagnosis, the performance of RDTs and microscopy should be regularly monitored to support appropriate treatment of malaria infections with effective antimalarials as part of the strategies to fight ART-R and attain the elimination targets.

## Data Availability

The data used in this paper are available and can be obtained upon request from the corresponding author.

## Abbreviations

ACT: Artemisinin-based combination therapy
AL: Artemether-Lumefantrine
aOR: Adjusted odds ratio
ART-R: Artemisinin partial resistance
CI: Confidence interval
CDC: Centers for Disease Control and Prevention
cOR: Crude odds ratio
CSS: Cross-sectional survey
DBS: Dried blood spots
DMFP: District malaria focal person
GMPD: Geometric Mean Parasite Density
IDs: Identification numbers
IPTp: Intermittent preventive treatment
IQR: Interquartile range
IRS: Indoor residual spraying
ITN: Insecticide-treated nets
LSM: Larval source management
MRCC: Medical Research Coordinating Committee
MSMT: Molecular surveillance of malaria in Tanzania.
NIMR: National Institute for Medical Research
NMCP: National Malaria Control Programme
ODK: Open Data Kit software
PCA: Principal component analysis
PO-RALG: President’s Office, Regional Administration and Local Government
RDTs: Rapid diagnostic tests
rRNA: Ribosomal Ribonucleic Acid
SMPS: School Malaria Parasitological Survey
SOPs: Standard Operating Procedures
WHO: World Health Organization
